# Meeting of the American Medical Association at St. Louis

**Published:** 1854-06

**Authors:** 


### EDITORIAL DEPARTMENT.

### *Meeting of the American Medical Association at St. Louis.*

We egre gate from the St. Louis daily papers such a record of the proceedings of the Association, at its late meeting, as we think will be interesting to our readers:

### First Day—Tuesday, May 2, 1854.

The Seventh Annual Meeting of the American Medical Association convened at 11 o'clock yesterday morning, at the Verandah Hall, pursuant to adjournment of the Sixth Annual Medical Session, held in 1853, in New York.

Dr. Usher Parsons, of Providence, Rhode Island, Senior Vice President of the Association, took the Chair and called the Convention to order, announcing that Presitents and Vice Presidents of past Conventions were entitled by courtesy to seats on the platform.

Dr. Edwin S. Lemoine, of St. Louis, took his place as Secretary.

The Secretary then read letters from Dr. Knight, President of the Association, announcing his unavoidable absence, and also a communication from J. G. Adams, M. D., announcing the presentation of the sixth volume of the Transactions of the Imperial Academy of Medicine at Paris, and the probability of opening a correspondence with that body.

The President said that the association having assembled were ready to hear from the Committee of Arrangements.

Dr. Washington, Chairman of said Committee, reported, and addressed the Convention, wecloming in the name of the Committee, the Delegates in attandce.

The President, after addressing the Convention appropriately, announced that the business first in order would be to call the roll of members of the association, beginning at the State of Maine.

The attendance was uuite large. Among the list of members from this State we notice Profs. James P. White, of Buffalo; Alden March, of Albany; Chandler R. Gilman, and H. Davis, of New York; Drs. Louis A. Sayre, Jas. L. Phelps, and others.

A report from the Committee on Publication was read and referred.

Also, the report of Dr. Francis Condie, Treasurer of Association, read and ordered to lie on the table until audited.

The Convention next received nominations by delegations from States, preparatory to going into an election for officers of the Association for the ensuing year.

The roll being called, the President announced the Convention to be fully organized.

A resolution fixing the time of the morning session at from 9 to 1 o'clock, and the afternoon session at from 3 to 5 o'clock, was introduced and adopted.

### AFTERNOON SESSION.

The Convention having been called to order, Dr. Parsons, Senior Vice President, read a very interesting address to the Association, which we hope to present to our readers on another occasion.

It was moved and ordered that the thanks of the association be returned to the President, and the address be referred to the committee on publication.

The report of the New York Committee on the Norwalk disaster, recommending that brief sketches of the History of those members of the Association who met an untimely death by that catastrophe, was adopted.

The letter from John G. Adams, requesting the previous volumes of the transactions of the Association for Presentation to the Academy at Paris, was then read again.

On motion it was agreed to comply with the requests contained in the said letter.

A communication was then received from the New Hampshire Medical Society, containing resolutions to the effect that no one should be allowed to become a member of the Association who countenanced any character of empiricism.

Dr. McIlvaine inquired if the set of volumes containing the transactions of the American Medical Association, had been transmitted to the Academy of

Medicine at Paris. He desired it might be done, that our medical literature might take that position in Continental Europe, to which its merits entitled it.

Dr. Atlee replied that the proper steps had been taken to secure the presentation of the volumes to the Paris Academy.

The President then announced that volunteer communications were in order, whereupon Dr. Grose offered a resolution declaring that all future elections of officers of the Association should take place immediately previous to the adjournment of the Convention, instead of immediately after its meeting.

The resolution being seconded, was ordered to lie on the table, for one year, according to the regulations of the Association, before being acted upon.

Dr. Gross offered another resolution, declaring it to be disorderly for any future committees of arrangements to prepare a costly supper, or dinner, for the entertainment of the members of the Association. Dr. Gross remarked in support of the resolution that he had the misfortune, perhaps, to live in a small city, where the profession was poor, and unable to go to any extravagant expenditure in the entertainment of the Association, in case it should ever honor his city by holding its meeting there. His resolution, he further said, was prospective in its application, and intended to make no reflection on any arrangements that had been made by the profession of this city.

A warm and spirited debate sprung up on the resolution, in which Drs. Coons, McPheeters and Barnes participated.

Dr. Coons approved of the resolution, and the objects it was intended to effect. The cause of science could not be promoted by preparing costly dinners at great expense. The committee of arrangements in this city have been at great expense and trouble to make preparations for the reception of the association, and he thought such efforts and receptions did not tend greatly to forward the ends of the association. (These remarks elicited some decided evidences of disapprobation.)

Dr. McPheeters deprecated the expediency of dragging such matters before the association.

A motion to lay the resolution on the table was lost.

An amendment was then offered by Dr. McPheeters, striking out the word "disorderly," and substituting a clause which modified the resolution so as to request future committees of arrangements not to prepare costly entertainments for the association.

Dr. McIlvaine desired to amend further by a clause prohibiting the use of liquor and tobacco. After some further discussion, Dr. Gross' resolution, with Dr. McPheeters' amendment, was carried.

The nominating committee then entered, and presented their report nominating the officers of the association for the ensuing year. Their report was unanimously received.

The following are the names of the officers thus nominated and elected:

*President*—Charles A. Pope, of Missouri.

*Vice Presidents*—E. D. Fenuer, of Louisiana; N. S. Davis, of Illinois; William T. Wragg, of South Carolina, and John Green, of Massachusetts.

*Secretaries*—Edwin S. Lemoine, of Missouri; Francis West, of Pennsylvania.

*Treasurer*—D. F. Condie.

The President then appointed a committee to wait on the officers, inform them of their election, and conduct them to their approrpriate seats.

Dr. Pope being absent on account of the sickness of his family, the senior Vice President took the chair.

It was moved then that Philadelphia be the place selected for holding the next Convention of the Association. Carried unanimously.

A series of resolutions were then reported from the committee on publication, to which an amendment was offered, providing a fee of three dollars should be required, of each member annually, to defray the expense of publishing the transactions of the Association, and that the name of any member refusing or neglecting to pay the same, should be erased from the list of permanent members. A spirited and interesting debate sprung up on the amendment.

Dr. Atlee supported the amendment, saying it was only asserting in theory, what had always been the practice of the Association. The opposers of the amendment asserted that absence, accident, or circumstances beyond the control of a member, might prevent him from paying his due, in which case, it would be manifestly unjust to strike out their names.

Dr. McIlvaine approved of the amendment: it would make members more prompt and punctual in the payment of their dues.

During the discussion, a motion that the resolutions be acted on separately was lost.

The resolutions were then further amended by providing that the Secretary, before erasing the name of a member should inform him of his indebtedness, and in this form passed *en masse*, by a unanimous vote.

A communication was then read from the Detroit Medical Society, requesting the Association to hold its next meeting in that city.

Dr. Atlee made some remarks in regard to the labors of a committee appointed by the Association at its meeting in Richmond, to take the necessary steps for providing a stone with a suitable inscription, to be placed on the tomb of Washington. Dr. Atlee said he had procured the design of an engraving by an American artist on Vermont marble, and only $400 were needed to secure the completion of the work. The engraving, of which a daguerreotype copy was exhibited, represented Hippocrates refusing to accompany Artaxerxes into Asia, as physician to the king's household. After the conclusion of Dr. Atlee's remarks the members gathered around him to contribute to the sum required to complete the work.

A motion was then made and unanimously carried that Dr. Charles Hooker be appointed Treasurer *pro tem.*, on account of the illness of the regular Treasurer.

A motion was carried that all resolutions shall be reduced to writing and signed by the author.

Dr. Pinkney, of the navy, was then introduced and read an eloquent address to the Association on the importance of union and concert in securing the exaltation of the medical profession.

At this moment Dr. Pope, the newly elected President, appeared in the hall and was conducted to the chair. He made a few brief and eloquent remarks to the Association. Honor, he said, was to be estimated according to the source from whence it was to derived. He esteemed his elevation to his present station as a mark of peculiar honor and confidence. He could have desired that it had been conferred on some one older aud worthier than himself. He yielded to no one, however, in an ardent devotion to, and a ceaseless love for his profession.

A communication was read from L. M. Kennett, inviting all the members of the Association to his residence. Also one from the Directors of the Missouri Institution for the Blind, requesting the members to visit that establishment.

A motion was made and carried that the nominating committee report to the association all the standing commiittees.

The President then invited the members to the residences of Drs. Moore, McPheeters and Reyburn.

On motion, the convention then adjourned, to meet on Wednesday, at 9 o'clock.

### MORNING SESSION.

Wednesday, May 3.

The Convention met at 9 o'clock, and after the reading of the minutes, which were slightly altered, they were adopted.

On motion, it was resolved that the meeting hereafter adjourn in the afternoon at 5 o'clock.

Dr. Atlee, of Pennsylvania, moved that the memorial to the Association from the American Medical Society of Paris be read. The motion carried, and the memorial was accordingly read, as follows:

To the American Medical Association:

We, the members of the American Medical Society of Paris, beg, through our delegates, to present the following memorial:

The National Association of the United States, has had its origin mainly from the consciousness of physicians of the low state of medical education in our country, and from the desire universally entertained by them, of elevating the standard of medical education and attainment of the medical profession.

We, by our sojourn abroad, from an intercourse with those educated here, have become more painfully conscious of our infirmities and deficiencies at home, and for this reason beg once more, to urge upon the Association the necessity of a change. While acknowledging, however, the superiority of education in Europe, we are far from desiring to arrive at equality by imitating their methods. We therefore beg to urge the following plans for the consideration of the Association:

That in each state there be appointed by the Medical Society of the state, a Board of Examiners, which board shall be chosen every year from members of the Society, and which shall perform its duties the following year, in the place and immediately before the sitting of the Society; that their examinations be public, and that any one whosoever may apply who shall be introduced by a member of the Society, and that no one can hereafter become a member of the state Medical Societies, nor of the American Medical Association, who has not the certificats of having satisfactorily passed such examination.

As to the qualifications to be required of the candidates, we do not think it advisable to enter into particulars. They should not, however, believe in any peculiar doctrines or methods—no certificates of attendance upon courses of lectures should be necessary, but solely the possession of the necessary amount of medical knowledge to practice their profession with safety and honor.

This plan in no way interferes with the established schools; its effect upon them could only be salutary. Students would attend those institutions where those branches of medical education that can only be acquired by attendance upon lectures, are best taught.

All of which is respectfully submitted to consideration of the Association.

DR. HAMMER, St. Louis,
DR. MURPHY, Cincinnati. } Committee.

Paris, March 21st, 1854.

The memorial was referred to the Committee on Publication.

The next business, as announced, was the receiving the reports of Special Committees. Several Committees were continued on motion, on account of the absence of the Chairmen, viz., Drs. Arnold, of Ga.; Wood, of N. Y.; Panche, of S. C.; Hooker, of Conn.; and Lapsley, of Penn.

It was moved that the report of Dr. Kane, of South Carolina, Chairman of the Committee on the Epidemics of South Carolina, Georgia and Alabama, not being entirely prepared, be referred to the Committee on Publication. Carried.

Dr. Mendenhall, of Ohio, read an abstract of a report on the Epidemics of Ohio, Indiana and Michigan, describing the geological features of those states, and the prevalent diseases during the years 1852 and 1853.

On motion the report was referred to the Committee on Publication.

Dr. Palmer, of Illinois, mentioned the convenient arrangements of the state of Michigan into districts, by which means ample facilities were offered for collecting statistics in reference to epidemics.

Dr. Atlee, of Pennsylvania, called on his brethren from all parts of the Union to give their aid to the Committees in gathering the requisite information in regard to the prevalent diseases of the country.

Dr. Holmes, of Missouri, read the Resume of a report on erysipelas. Referred to the Committee on Publication.

Dr. Fenner, of Louisiana, Chairman of the Committee on the Epidemics of Louisiana, Mississippi, Texas and Arkansas, read a report on that subject, describing the effects and operations of yellow fever and cholera. It was ordered that the report, when completed, be referred to the Committee on Publication.

On motion of Dr. Mussey, the regular order of business was suspended, to allow Dr. Linton, of St. Louis, to express his views on the subject of yellow fever. Prof. Linton's opinion differed from the generally received opinion in regard to the causes which produce the fever. He repudiated the idea of malarious influence in producing the disease, and attributed it to heat and northern blood.

On motion, Dr. Linton was requested to write out his views, and send them to the Committee on Publication.

Dr. Brainard asked leave for further time in preparing his report. Granted.

Dr. Davis read an abstract of a report on Typhoid Fever.

The report was referred.

The report of Dr. Donaldson, of Baltimore, on the use of the microscope, was ordered to be referred to the Committee on Publication, in accordance with a communicated request from Dr. Donaldson, who was absent.

The report of Dr. Howard, of Ohio, being called, his death was announced by a colleague.

The report of Dr. Cabell, of Virginia, on Medical Education, was referred.

Dr. Pope, Chairman of the Committee on Prize Essays, reported that Professor Daniel Brainard, of Chicago, Illinois, was the successful competitor for the prize awarded for the best essay; subject, "Treating and Uniting Fractures."

The announcement was greeted with applause, and on motion of Dr. McPheeters, Dr. Brainard was invited to the stand to explain his new mode of treating and uniting fractures.

Prof. Hooker, of Conn., Treasurer pro. tem., called the attention of the members to the duty of paying the annual assessment, and recommended the appointment of committees in each state to attend to the sale and distribution of the Transactions of the Association.

Dr. Elbert, of Iowa, introduced resolutions providing for the appointment of a committee whose duty it shall be to suggest amendments to the Constitution, and recommending the election of officers, and the selection of a place of meeting, by ballot, without the intervention of the Nominating Committee. Lost.

Dr. Guthrie, of Kentucky, offered a resolution approving the recommendation of the Secretary of the Treasury to Congress, that the duty on drugs not produced in this country, be reduced. The resolutions were adopted.

Dr. Johnson, of St. Louis, produced a letter from Dr. Williams, calling up resolutions, introduced a year previous, providing for the appointment of a standing committee, to gather facts connected with the history of eminent members of the Association, and publish them, with brief sketches of their lives, in the Transactions of the Association. The resolutions were passed.

"*American Medical Biography, for the American Medical Association, assembled at St. Louis:*

"Mr. President,—At the last meeting of the Association in New York, I presented the following preamble and resolution through my friend Dr. Stewart, of New York, which, with some amendments, were laid on the table.

"As we are constantly called upon to deplore the ravages of death among the meritorious and worthy members of our profession throughout the United States,

"Resolved, That a standing committee be appointed by this Association to procure memorials of the eminent and worthy dead among the distinguished physicians of our country, and present them to this Association for publication in their Transactions.

"I now beg leave to call up the resolutions through my friend Dr. J. B. Johnson, of St. Louis. The medical biography of our country is intimately related to the history of it, as the lives of eminent men are identified with the history of the times in which they lived. In the United States have been, and are to be found, medical men whose lives and actions are ornaments to human nature, and whose brilliant career in the cause of humanity and science, reflect honor and dignity upon our country. We suffer nothing in this respect in comparison with the learned and eminent physicians in Europe. The profession of Medicine contains more learned and distinguished men than any other profession or calling, and some memorial of their lives and actions should be presented to the world in a more durable form than the periodical journal of the day, and particularly of the newspaper press. A more permanent and proper place for the publication of such memorials would be in the Transactions of the American Medical Association. And without disparagement to any other articles which have heretofore been published in the Transactions, such memorials would be read by the surviving members of the profession with great interest and improvement. Short biographies need take up but little room in the publication—and this objection to the proposed movement would be thus obviated.

"We are constantly noticing that death spares no ranks or conditions of men. Those who contend most skillfully against his insatiate ravages, themselves fall victims to his all-conquering sword. Within a very short space of time, we have been called to lament the deaths of Dr. Nathaniel Chapman, the former President of this Association; of Drs. Samuel G. Morton, Wm. E. Harner, Isaac Parish, G. S. Pattison, J. Kearney Rodgers, Daniel Drake, the great Medical Pioneer of the West; Samuel McClellan, Amos Twitchell, Abiel Pierson, G. C. Shattuck, Archibald Welch, and very many others which time will not permit me to enumerate.

"This is not the place to speak their eulogies; some permanent notice of them and of many others who have recently died, should be published in the transactions of this Association, where the useful improvements and discoveries of the living should be recorded, and the memories of the worthy dead should be presented. STEPHEN W. WILLIAMS,

Lavina, Winnebago Co., Ill. Late of Dufield, Mass.

Dr. McIlvaine, of Ohio, offered a resolution declaring that the habit of reading lectures to medical classes, is a miserable way of teaching, and inimical to medical progress. Laid on the table.

Dr. Ramsay offered a resolution providing for the abolishment of the Nominating Committee. Laid on the table for one year.

A resolution was adopted providing for the appointment of a Committee whose duty it shall be to inquire whether any irregular practitioners are connected with this Association.

A communication was presented by Dr. Blatchford, of New York, from Dr. Spore, of the same State, on the subject of Hydrophobia, and its connection with the seasons. A resolution appointing a committee on Hydrophobia, with Dr. Blatchford as Chairman, was adopted.

Dr. McDowell, of St. Louis, explained an attack made by him on an instrument invented by Professor Smith, of Boston, for performing the operation of Lithotomy. Dr. McDowell desired the appointment of a committee to investigate the matter, and give the credit of the invention to whom it is due. A resolution to that effect was laid on the table.

Dr. Ramsay, of Tennessee, offered a resolution, making it the duty of a committee appointed according to a previous resolution, to inquire into the charges made against some members of this Association of using proprietary medicines. Adopted.

A resolution was then passed, appointing a committee to inquire into the subject of insanity, as it prevails in this country, and report on the same.

The Convention then adjourned.

[Of the proceedings of the afternoon of the second day we have, unfortunately, no record. The proceedings of the third day will be read with interest by all who desire the prosperity of the Association. The action of the Committee on Nomination, of which Prof. J. P. White, of this city, was Chairman, was the subject of much excited debate. But we doubt not that our readers will rejoice with us that a blow has been struck at that hierarchy which had assumed the special charge of the affairs of the Association, and claimed an undue influence in its operations.]

THIRD DAY—THURSDAY.

The Association convened at nine o'clock, A. M. Dr. Pope, President, in the chair.

On motion, the regular order of business was suspended for the purpose of filling the vacancy in the Nominating Committee from Iowa.

On motion, Dr. Mcgingan was chosen to fill the vacancy.

The minutes of Wednesday's proceedings were read, and after a few unimportant amendments, were adopted.

Dr. McPheeters stated to the Association that arrangements had been made with the different lines of travel from the city, to convey the members of the Association to their homes, free of charge; and that all the lines and companies had agreed to the arrangement, excepting the New York and Hudson River Railroad Company.

The Secretary now read a communication from the Chairman of the Nominating Committee, requesting that body to meet. Also, a communication tendering the hospitality of the city of Burlington, Iowa, to those members who return by the Upper Mississippi.

Dr. Atlee offered the following resolution, which carried:

*Resolved*, That it shall be the duty of the Publication Committee to append to each volume of the transactions hereafter published, a copy of the Constitution of the Association.

The following resolution, offered by Dr. Gross, was also carried, and Dr. Gross was appointed by the Chair the Committee designated:

*Resolved*, That a Committee of one be appointed by the Chair to inquire into the causes which obstruct the formation and establishment of our National Medical Literature, and to report the subject at the next annual meeting of this Association, or as soon thereafter as practicable.

The following communication from the Engineer and Superintendent of the Pacific Railroad, was read by the President:

Dr. Washington, Chairman and Com. of Arrangements;

Dear Sir; I am authorized by the Directors of the Pacific Railroad to offer to your Committee the use of the road, at your convenience, in case you should desire to show to the members of the Medical Society that are expected to meet here, the country in the neighborhood of St. Louis, and go a few miles out on what we hope will in time be *the* road to the Pacific Ocean.

If you will give me one day's notice, I will send out a special train, at such an hour as may suit your arrangements. Very respectfully,

THOS. S. O'SULLIVAN, Eng. and Sup't.

On motion of Dr. Atlee, a vote of thanks was extended by the Association to the Directors of the Pacific Railroad.

The Chairman of the Committee of Arrangements moved that the Association accept the invitation of the Company—with the information that 10 o'clock A. M. on Friday would be a convenient hour for the excursion to be made, as this would allow opportunity for delegates, who wished to leave the city in the afternoon, to be in season for boats, trains, &c.

Dr. J. Berrien Lindsley offered the following resolution, which, on motion, was referred to the Committee on Medical Education, with instructions to report at the next Annual Meeting of the Association:

*Resolved*, That this Association earnestly recommend to the few Western schools which still retain the rule of making four years' practice equivalent to one term at College, the abrogation of said rule, as holding out a strong inducement and temptation to young men to enter upon the practice of medecine with little or no preparation.

Dr. Paul F. Eve, of Nashville, Tenn., submitted a resolution, which, after amendment, as follows, was carried:

*Resolved*, That a committee of three be appointed by the Chair, to report at the next meeting of the Association, the best means for preventing the introduction of disease by emigrants into our country.

The Chair appointed Drs. Dickson, Griscam and E. D. Fenner, a committee.

Dr. Linton of St. Louis, offered the following, which was also referred to the above named committee:

*Resolved*, That in the opinion of this Association, quarantine establishments afford no protection to states and cities against the invasion of epidemics such as cholera and yellow fever.

Dr. Penn offered a resolution to the following effect:

*Resolved*, That the members of the Committee of Arrangements who are not members of the Medical Association, be invited to take seats in the Association, as members by invitation.

Which was carried.

Dr. Jayne arose and offered a resolution in relation to the memorial from Drs. Hammer and Murphy, of the American Medical Society at Paris, to the American Medical Association, which was, at a previous meeting, referred to the Committee on Education.

*Resolved*, That the memorial from Drs. Hammer and Murphy be withdrawn from the Committee on Education.

Dr. Jayne spoke at some length regarding the memorial referred to and the object of his resolution.

He stated that such a document implied the inefficiency of American Professors of Medicine to give students a complete and thorough scientfic education, and cast unjust and shameful reflections upon the capacity of medical men in this country. He contended that such a document, being so disposed of, appearing in the published proceedings of the American Medical Association, to be read in all parts of the Union, would confer a lasting disgrace on the judgment and deliberations of that body, and on the whole Faculty in this country. He warmly supported the object of the resolution, and submitted it.

Dr. Atlee remarked in this connection, that he knew something of the motives of the young men who wrote it, and was convinced that it was dictated by the best intentions, and without any desire on the part of the authors, to underrate the members of the profession, or medical science in this country.

Dr. Elbert entered warmly into the discussion, and remarked that the Association should act upon the document as it appeared. That it spoke for itself, and that the Association had nothing to do with intentions outside of the language of the document.

Dr. McIlvaine wished the objection to the document more clearly specified than he had yet heard it. He believed it to have been sent in good faith.

Dr. Edgar then made some statements about the loose and incompetent education received by some Physicians in this country in certain schools, before they were considered qualified and permitted to enter upon the practice of the profession. He instanced two young men who had commenced the study of medicine in his office, and whom after remaining six or eight months, be candidly told that they would never make competent Physicians. The young men left, and in eighteen months and or years from that time they were regular practicing physicians, having passed through some school which he did not name. He contended that some protection was necessary. He said, moreover, that an effort was made some time ago in this state to establish a Board of Censors, under whose examination every student should be required to pass before entering upon the duties of the profession, but that it failed, the physicians arising *en masse* against it, with the single exception of Dr. Linton.

After some more discussion the memorial was withdrawn from the Committee of Education and laid on the table.

A communication was read from Dr. Peebles, chairman of the Committee on Epidemics of Virginia and North Carolina, requesting to be excused from the committee. The request was granted.

The Secretary stated that a document had been received from Dr. Phelps, of New York, purporting to be an abstract of a paper explaining the relation between miedicine and religion.

On moton the regular business was suspended to give place to the reading of the report of the Nominating Committee, of which the following is a copy:

### REPORT OF THE COMMITTEE OF NOMINATIONS.

The Committee on Nominations, in fulfilling the duty imposed upon them, recommend the continuance of several of the special committees previously created, and the appointment of some new ones. They, therefore, submit the following list of Chairmen of special committees, with the subjects to them committed:

Dr. Worthington Hooker, of New Haven, Connecticut, on epidemics of New England and New York.

Dr. John L. Atlee, of Lancaster, Pa., on epidemics of New Jersey, Pennsilvania, Delaware and Maryland.

Dr. D. J. Cain, of Charleston, S. C., on epidemics of South Carolina, Florida, Georgia and Alabama.

Dr. W. L. Sutton, of Georgetown, Ky., on epidemics of Tennessee and Kentucky.

Dr. Thos. Reyburn, of St. Louis, Mo., on epidemics of Missouri, Illinois, Iowa and Wisconsin.

Dr. Geo. Mendenhall, of Cincinnati, Ohio, on epidemics of Ohio, Indiana and Michigan.

Dr. E. D. Fenner, of New Orleans, La., on epidemies of Mississippi, Louisiana, Arkansas and Texas.

Dr. James Jones, of New Orleans, La., on the mutual relations of yellow and bilious remittent fever.

Dr. D. V. Condie, of Philadelphia, Pennsylvania,— On the causes of Tuberculous Disease.

Dr. Jos. Leidy, of Philadelphia, Pa, on diseases of the Parastic Origin.

Dr. A. P. Merrill, of Memphis, Tenn., on the Physiological Peculiarities and Diseases of Negroes.

Dr. Jos. N. McDowell, of St. Louis, Mo., on Statistics of the operation for the Removal of Stone in the Bladder.

Dr. F. P. Porcher, of Charleston, S. C., on the Toxicological and Medicinal Properties of our Cryptogamic Plants.

Dr. Daniel Brainard, of Chicago, Illinois, on the Constitutional and Local Treatment of Corcinoma.

Dr. Geo. Engleman, of St. Louis, Mo., on the Influence of Geological Formations on the Character of Disease.

Dr. Henry Taylor, of Mount Clemens, Mich., on Dysentery.

Dr. Horace Green, of New York, on the use and effect of applications of Nitrate of Silver to the Throat in Local or General Disease.

Dr. P. C. Gooch, of Richmond, Va., on the administration of Anaesthetic Agents, during Parturition.

Dr. Chas. Hooker, of New Haven, Conn, on the Diet of the Sick.

Dr. E. R. Dabney, of Clarksville, Tenn., on certain forms of Eruptive Fevers, prevalent in Middle Tennessee.

Dr. Sanford B. Hunt, of New York, on the Hygrometrical State of the Atmosphere in various localities, and its influence on health.

Dr. Frank H. Hamilton, of Buffalo, New York, on the frequency of deformities in fractures.

Dr. M. M. Pallen, of St. Louis, Mo., on diseases of the Prostate Gland.

Dr. H. A. Johnson, of Chicago, Ill., on the Excretions as an index to the Organic Changes going, on in the system.

Dr. Leroy H. Anderson, of Sumpterville, Ala., on Typhoid Fever and its complications as it prevails in Alabama.

Dr. W. H. Byford, of Evansville, La., on the Pathology and Treatment of Scrofula.

Dr. N. S. Davis, of Chicago, Ill., on the Nutritive Qualities of Milk, and the influence produced thereon by pregnancy and menstruation in the human female, and by pregnancy in the cow, and also on the question whether there is not some mode by which the nutritive constituents of milk can be preserved in their purity and sweetness, and furnished to the inhabitants of cities in such quantities as to supersede the present defective and often unwholesome method of supply.

Dr. E. B. Haskens, of Clarksville, Tenn., on the Microscopical Investigations of Malignant Tumors.

Dr. Geo. K. Grant of Memphis, Tenn., on the Sulphate of Quinia as a Remedial Agent in the treatment of Fevers.

Dr. R. R. McIlvaine of Cincinnati, Ohio, on the Study of Pathology at the Bedside.

Dr. E. S. Cooper of Peoria, Ill., on Orthopedic Surgery.

Dr. Andrew F. Jeter of Palmyra, Mo., on the Modus Operandi of the Envenomed secretions of healthy Animals.

Dr. Sam. M. Smith, of Columbus, Ohio, on Insanity.

Dr. Rene La Roche of Philadelphia, Penn., on the Jaundice of Yellow Fever in its Diagnostical and Prognostical Relations.

Dr. Charles Chandler of Rocheport, Mo., on Malignant Periodic Fevers.

Dr. S. B. Chase, of Portland, Maine, on Typhoid Fever in Maine.

Committee on Plans of Organization for State and County Societies — A. B. Palmer, M. D., Michigan; R. R. McIlvaine, M. D., Ohio; D. L. McGugin, M. D., Iowa; E. R. Peaslee, M. D. New Hampshire; Thos. Lipscomb, M. D. Tennessee.

Committee on Medical Literature—Robert J. Breckenridge, M. D., Kentucky, Chairman; A. A. Gould, M. D., Mass.; D. L. McGugin, M. D, Iowa; J. B. Flint, M. D., Ky.; O. M. Langdon, M. D., Ohio.

Committe on Medical Education—Wm. H. Anderson, M. D., Alabama; A. Lopez, M. D., do; Andrew Murray, M. D., Michigan; A. Ramsay, M. D., Tennessee; R. D. Ross, M. D.

Committee on Prize Essays—Rene La Roche, M. D., Pennsylvania; Isaac Hays, M. D., do; Alfred Stille, M. D., do; J. B. Biddle, M. D., do; Geo. W. Norris, M. D., do; Joseph Carson, M. D., do; Joseph Leidey, M. D., do;

Committee of Arrangements — Isaac Hays, M. D., Pennsylvania; G. Emerson, M. D., do; Wilson Jewell, M. D., do; Alfred Stille, M. D. do; Francis West, M. D., do; Wm. V. Keating, M. D., do.

Committee on Publication—Pliny Earle, M. D., New York; D. Francis Condie, M. D., Pennsylvania; E. S. Lemoine, M. D., Missouri; A. March, M. D., New York; E. H. Davis, M. D., do; C. R. Gilman, M. D., do.

After the reading of the report, Dr. Reyburn moved its adoption, excepting that portion referring to the Committee on Publication, in the following resolution:

*Resolved*, That the said report be adopted, with the exception of that portion which refers to the Committee on Publication.

He remarked, in support of the resolution, that he saw no reason why the publication of the proceedings of the Medical Society should be changed from Philadelphia to New York, as was evidently intended by placing the Philadelphia portion of the Committee in minority. He did not think the committee of New Yorkers would attend to the business any better, and as the old committee had always performed their duty, he did not see the necessity of a change.

Dr. Jayne remarked that it was unprecedented in the annals of the Association that any part of the report of the Nominating Committee should be stricken out. He said that the Association might as well appoint a permanent President as a permanent Committee of Publication. He thought it was but justice to that committee to take the labor and responsibility off their hands, as they had labored long and arduously for the Association, and it had positively no right to entail upon any committee such constant labor. He was, therefore in favor of changing the committee and place of publication, and thus dividing the labor consequent upon the duties.

Dr. Steward contended that the sole reason for the change was that the, gentlemen in New York wanted the publication there; but as their wants were not supported by sufficiet reasons, the Association should not take cognizance of them; that it ought, on the other hand, to retain the old committee, as those gentlemen have not declined, and no argument has been advanced why they should be discharged.

We have no right, even if we had the power, to discharge a faithful officer. The gentlemen comprising the committee have labored faithfully and efficiently in the discharge of their arduous duties, and have subscribed liberally from their own pockets, in the event of a scarcity of funds. One gentleman paid $10 for his copy of the proceedings. Dr. Steward was, therefore, not in favor of taking the publishing out of the hands of those who had proved themselves competent, and warmly devoted to the interests and objects of the Association.

Dr. Paul F. Eve wished the nomination made by the nominating committee confirmed, as they had generally been accepted by the Association. He saw no reason why the change should not be made, and he had understood that $1000 or $1200 had been consumed last year in the getting up of one gentleman's contributions.

It was, however, explained that the Association had agreed to the expense in this case.

Dr. Paddock asked the question, why the poblication should not be given to some city in the West? St. Louis, for instance.

Dr. McPheeters answered that St. Louis did not want it, and that it could not be done here so cheaply, nor so well, as in the East.

Dr. Paddock contended that the subject was being discussed upon the wrong ground. He did not see that the question necessarily involved any censure on Philadelphia. He did not find fault with the action and performance of the committee. The character of the medical profession in Philadelphia has stood high enough since the days of Dr. Rush.

Dr. Paddock said that he was a Mississippi Valley man, and wished to do what was proper, without reference to any one else. It is not our business to show that there has been a fault, nor advance arguments against the Philadelphia Publication Committee. After continuing his remarks for some time a gentleman asked the Chairman if he had occupied the fifteen minutes allowed to members, when the President replied in the negative; the interrogator said he was very sorry.

Dr. Paddock then continued. The Committee report in favor of publishing in New York. The profession in New York, are as anxious to advance the interest of the Association as in Philadelphia, and I do n't see why they should not have an opportunity of manifesting professional pride and skill.

Dr. Herrick, of Ill., stated that he was one of the Committee of Nomination, and wished to state some reasons why the change was made. He did not see that it would materially affect the matter whether a majority of the committee were in Philadelphia or New York. They considered it a matter of duty to make the change. As it is a great National Association we should endeavor to change the labor, as well as the honor, from one portion of the Union to another. If an office of labor, it is wrong to impose on one portion of the Association—if a place of profit, the benefits should be distributed.

The Publishing Committee have authority to publish or not any communication that may be laid before them, and that gives power to one set of individuals to bring their works before the public before another. This advantage should not be allowed. I was for changing the majority of the committee to any place away from Philadelphia. The foregoing are the principal reasons he advanced for the change.

Dr. McPheeters made a short reply in favor of the resolution.

Dr. Sayre now made a motion that the matter be referred to a committee of the whole, which carried and the Association passed into such a committee.

On motion, Dr. Elbert took the chair, and the discussion again commenced with renewed ardor.

Dr. Davis, of Chicago, spoke at some length. He said that the change implied no censure. Dr. Condie is still retained on the committee, also the secretary, Dr. West. There have been added to the council two or three members from New York. Censure will accumulate. No set of men can hold an office without censure. Let the censure move around and we will save any one from unjust labor and reproach.

The discussion lasted two or three hours, and was warmly supported. The committee of the whole at length reported back the amendment of Dr. Reyburn, which was adopted. The committee then rose and the Association adjourned.

### AFTERNOON SESSION.

The Association convened at three o'clock; Dr. Pope in the chair.

A resolution was offered by Dr. Storer in relation to permanent members, which was unanimously carried.

The Treasurer said that he had an announcement to make for the benefit of the friends of the Association. He stated that in the morning he had taken a ten guilder piece, mistaking it for five dollars, and had in his possession a three dollar doubtful bill, which the brokers refused, and that one of the members had taken a receipt for three dollars without paying the money. He advocated the rectifying of these mistakes.

A resolution was offered to the effect that W. S. Maus, M. D., of Pekin, Illinois, be elected a permanent member—which was carried unanimously.

Dr. Atlee offered the following resolution, which was carried:

*Resolved*, That this Association earnestly recommend to their medical brethren in those states in which societies do not exist, the immediate organization of State and County Medical Societies.

Dr. Ramsey offered a resolution, which, on motion of Dr. Coons, was laid on the table.

Dr. Breckenridge offered a resolution to the following effect:

*Resolved*, That the papers and documents of the Association shall hereafter be the exclusive property of the Association, which, as amended by Dr. Smith, was carried.

Dr. Phelps, of New York, again requested to read the document which he presented in the morning, and the reading of which was deferred to allow the Committee of Nominations to make their report.

The document wss referred to a Special Committee, to be appointed by the Chair. The President named Dr. Atlee, Dr. Sayre and Dr. March, to serve on the Committee.

A motion was now made to proceed to the regular business which was carried.

Dr. Eve moved that the matter relating to the report of the Committee on Nomination, and the blank occasioned by the amendment offered by Dr. Reyburn, which was adopted in Committee of the Whole, be referred back to the Nominating Committee for the purpose of filling up the blank, which was lost.

The question then came up upon adopting the original report of the Committee of Nominations. A discussion arose, participated in by Drs. Breckenridge, Atlee, Sayre, Storer, McDowel, White, and others. It was in character much the same as that of the morning, becoming very animated, and at times personal feeling and sectional jealousy were evinced.

The original report of the Committee was at length adopted.

After the vote was announced the delegation from Philadelphia, through Dr. La Roche, announced that they would take the responsibility of tendering the resignation of Dr. Condie, of Philadelphia, Treasurer of the Association. After some little discussion the resignation of Dr. Condie was accepted.

Dr. West, of Philadelphia, one of the Secretaries, then tendered his resignation, and the question being upon accepting it, it was lost.

Dr. Breckenridge, of Kentucky, then offered the following resolution, which was carried:

*Resolved*, That hereafter the majority of the Committee on Publications shall be selected from the physicians of that city in which the Association may annually meet.

A vote of thanks was then unanimously returned to Dr. Condie for the able, zealous and impartial manner with which he had discharged his duties as Treasurer.

A resolution was reported to amend the constitution, which provides that its annual meetings shall be held on the first Monday in May, and substitute the second Monday. The resolution, under the rule, lies over for a year.

Dr. W. H. Byford offered a resolution of thanks to Dr. Pinkney, of the United States Navy, for the able address delivered before the Association on the first day of its session, which was unanimously adopted.

Dr. Atlee offered a resolution tendering the thanks of the Association, and of the individual members, to the citizens of St. Louis for their hospitality and kindness; also, to the Directors of the various Railroads and officers of steamboats for the generous manner in which they have tendered their kind offices, which was adopted.

Dr. White moved that a vote of thanks be extended to the late Publishing Committee for their best endeavors to serve the Association, which was unanimously adopted.

A resolution offered by Dr. W. W. Hitt, about alcoholic drinks, was adopted and referred to the Committee on Nominations.

A resolution offered by Dr. C. B. Hughes, relating to specialty practice in Surgery, was on motion laid on the table.

Dr. White, from Nominating Committee. reported to the Association the name of Dr. Blatchford, of New York, as Treasurer, in place of Dr. Condie, resigned.

Dr. Blatchford declined acting in that capacity, and the committee subsequently reported the name of Dr. Wood of New York. They also reported a special committee on Epidemics, for the states of Virginia and North Carolina, with Dr. Haskins as chairman; and also, the resolution relative to alcoholic drinks was reported back by them, referring it to a special committee, consisting of Dr. Mussey.

Dr. W. S. Edgar offered a resolution in regard to the compounding of medicine, and recommending apothecaries to use different colored paper in putting up poisonous drugs, with an appropriate stamp upon it, in contradistinction to other medicines.

